# Metabolic biomarkers and cardiometabolic risk among night shift workers: evidence from night shift workers in Europe

**DOI:** 10.1093/eurpub/ckag101

**Published:** 2026-07-09

**Authors:** Barbara N Harding, Caisa Laurell, Jesse D Thacher, Gemma Castaño-Vinyals, Anastasiia Snigireva, Anne Helene Garde, Kirsten Nabe-Nielsen, Maria Albin, Manolis Kogevinas, Karin Broberg

**Affiliations:** College of Population Health, University of New Mexico, Albuquerque, NM, United States; Institute of Environmental Medicine, Karolinska Institutet, Stockholm, Sweden; Institute of Environmental Medicine, Karolinska Institutet, Stockholm, Sweden; Department of Laboratory Medicine, Lund University, Lund, Sweden; Barcelona Institute for Global Health (ISGlobal), Barcelona, Spain; Universitat Pompeu Fabra (UPF), Barcelona, Spain; CIBER Epidemiología y Salud Pública (CIBERESP), Madrid, Spain; Institute of Environmental Medicine, Karolinska Institutet, Stockholm, Sweden; The National Research Centre for the Working Environment, Copenhagen, Denmark; The National Research Centre for the Working Environment, Copenhagen, Denmark; Department of Public Health, University of Copenhagen, Copenhagen, Denmark; Institute of Environmental Medicine, Karolinska Institutet, Stockholm, Sweden; Department of Laboratory Medicine, Lund University, Lund, Sweden; Barcelona Institute for Global Health (ISGlobal), Barcelona, Spain; Universitat Pompeu Fabra (UPF), Barcelona, Spain; CIBER Epidemiología y Salud Pública (CIBERESP), Madrid, Spain; Institute of Environmental Medicine, Karolinska Institutet, Stockholm, Sweden; Department of Laboratory Medicine, Lund University, Lund, Sweden

## Abstract

Circadian disruption resulting from night shift work has been associated with cardiometabolic diseases, but the underlying biological pathways remain insufficiently understood. We analyzed data from the EPHOR-NIGHT cohort, including questionnaires, clinical assessments [body mass index (BMI), blood pressure (BP), waist/hip circumference], and plasma metabolites in blood samples (*N* = 860; day shift workers *n* = 362, night shift workers = 498) from Sweden, Spain, and Denmark. We applied multivariable linear regression to examine differences in cardiometabolic risk factors and metabolite levels between shift workers and examined associations between metabolites and cardiometabolic risk factors. Night shift work was associated with higher mean systolic BP (β = 1.89, 95%CI 0.00, 3.79 mmHg), higher mean BMI (β = 1.14, 95%CI 0.44, 1.84 kg/m^2^), and higher odds of hypertension (OR = 1.38, 95%CI 1.00, 1.89) and overweight/obesity (OR = 1.37, 95%CI 1.02, 1.82), compared to day shift work. Associations were stronger among women in sex-stratified analyses. Night shift workers had metabolic alterations, with lower fractions of polyunsaturated fatty acids, higher fractions of mono-unsaturated and saturated fatty acids, and higher levels of amino acids isoleucine, valine, and phenylalanine. These changes remained after multiple adjustments, including diet. Effects were more pronounced for those working more consecutive night shifts, more night shifts per week, and for permanent night schedules versus rotating shifts. The night shift-related metabolites were associated with higher BMI and higher blood pressure. Night shift work was associated with specific metabolic alterations linked to increased cardiometabolic risk. Our results suggest that reducing night shift intensity and consecutive nights may help mitigate these adverse effects.

## Introduction

Approximately 12–20% of the workforce in Europe is engaged in night shift work [1, 2]. Night shift work disrupts normal biological circadian rhythms [3] regulated by a central “clock” in the brain that regulates the rhythmical release of melatonin [4–6]. Under normal circumstances, the central clock synchronizes peripheral tissue clocks, including the liver, adipose tissue, and gut, which are also central to the metabolism of glucose and fat [7].

Circadian rhythm disruption has been associated with adverse effects on cardiovascular health, the leading cause of death worldwide [8]. Blood pressure, markers of obesity, and metabolites are early risk factors for further cardiometabolic disease [9], and they are important for improving and maintaining cardiovascular health. Compared to day shift workers, night shift workers have been shown to have a higher risk of developing hypertension and being overweight [10, 11], and increased prevalence of all-cause cardiovascular disease (CVD) and cardiometabolic disorders [12], including metabolic syndrome [13, 14] and type 2 diabetes [15]. Moreover, changes in metabolites such as cholesterol, fatty acids and amino acids [4, 14], and impaired glucose tolerance [4, 16], have been associated with night shift work.

Further research is needed to clarify mechanisms and dose-response patterns, including co-exposures, such as metals cadmium and lead [17, 18] that are linked to both circadian disruption and increased risk of CVD. Against this background, we undertook the present study to examine associations between night shift work and cardiometabolic risk factors and associated biomarkers in the EPHOR-NIGHT cohort, with a particular interest in whether the frequency, intensity, or duration of night shift work influenced the associations.

## Methods

### Study design

The EPHOR-NIGHT study is a longitudinal study; however, the present analyses are based on cross-sectional data collected at the baseline visit. Baseline data (e.g. demographics, lifestyle characteristics, diet, and sleep) collected through questionnaires, were combined with data on anthropometry, blood pressure, and plasma metabolites collected on one day during five continuous days of monitoring. The study design is described in detail in Harding *et al.* [19].

### Study population

The EPHOR-Night cohort was composed of 564 night shift workers (the exposed group) and 373 day shift workers (the unexposed group), with approximately equal proportions of night and day workers within each workplace. All participants were between the ages of 20–65 years. Participants in this study were sampled from three centers: Barcelona, Spain (bus workers and healthcare workers), Stockholm, Sweden (healthcare workers), and various locations in Denmark (healthcare workers).

### Night shift exposure

A night shift worker in our sample was required to work a minimum of two consecutive night shifts per week and have worked night shifts for more than three years prior to enrolment. However, due to difficulty in enrolling a sufficiently large population, a small portion of the sample had fewer years of prior night shift work (*n* = 54, 10% of the night shift group). A night shift was defined as a shift that involved working ≥4 h of the shift between 00:00 h and 06:00 h. For day shift workers, workers were not allowed to be currently working night shifts, nor to have worked night shifts for the prior three years at the time of enrolment. A day shift was defined as a shift that involved working ≥4 h of the shift between 07:00 h and 18:00 h (shift must start at 06:00 h or later). Information on the history of night shift work duration, which included the total number of years where participants had been engaged in night shift work prior to enrolment, was additionally collected. Permanent night shift workers worked only night shifts. Rotating shift work with nights was defined as those who rotated through multiple shift types, including night shifts. Information on night shift intensity (average night shifts per week) and the number of consecutive night shifts (average number of night shifts worked back-to-back before a break or change in shift schedule) was collected from questionnaires.

### Blood sample collection

Venous blood samples were collected at the workplace between 6:00 and 10:00 h for most participants (80%) after two consecutive night shifts for the night shift workers and two day shifts for day shift workers. For plasma metabolite measurements, EDTA blood tubes (Greiner Bio-One, Kremsmünster, Austria) were kept for 10 min before the plasma was separated at 2000×*g* for 10 min. The plasma was collected from the tubes and frozen at −80°C until further analysis. For measurements of concentrations of the metals lead and cadmium, performed in the Spanish and Swedish subsamples, blood was collected in trace elements tubes containing sodium heparin (Becton Dickinson, Plymouth, United Kingdom) and stored at −20°C prior to the analysis.

Fasting samples in the Swedish subsample were collected between 7:00 and 9:00 h on another day after a night with restriction of food intake and rest. For fasting blood glucose measurement, blood was collected in FC Mix tubes containing sodium fluoride, Na_2_EDTA, citric acid, and sodium citrate (Greiner Bio-One), and for hemoglobin A_1c_ (HbA_1c_) blood was collected in EDTA tubes (Greiner Bio-One). For both latter analyses, blood tubes were transported to the hospital laboratory of the Karolinska University Hospital the same day and analysed.

### Cardiometabolic outcomes

The following cardiometabolic outcomes were included: (i) systolic (SBP) and diastolic blood pressure (DBP) and a measure of hypertension, (ii) height (m), weight (kg) to calculate the BMI [weight (kg)]/[height (m)^2], (iii) waist circumference (cm) and hip circumference (cm) to calculate WHR (waist circumference/hip circumference), (iv) plasma metabolites including cholesterol, apolipoproteins, lactate, fatty acids, glucose and amino acids, (v) fasting glucose (Swedish subsample, *N* = 156), (vi) fasting HbA_1c_ (Swedish subsample, *N* = 169) (Flow chart in Fig. S1).

SBP and DBP were assessed from averaging the second and third (discarding the first) of three seated readings using an automated blood pressure device (OMRON MT 10 IT). A participant was considered to have hypertension if they met any of the following criteria: (i) SBP ≥130 mmHg, (ii) DBP ≥80 mm Hg [20], or (iii) hypertensive medication used based on information from the baseline questionnaire. The WHO guidelines [21] were used to categorize BMI into underweight (<18.5), normal (18.5–24.9), overweight (25–29.9), and obese (≥30) and then to create binary groups combining underweight/normal compared to overweight/obese; and WHR into abdominal obesity (≥0.90 for men and ≥0.85 for women) [22].

Total cholesterol, high- and low-density cholesterol (HDL/LDL), apolipoprotein A and B, total triglycerides, fatty acids, amino acids, glucose and lactate were measured in plasma with the Nightingale health platform (Nightingale Health, Helsinki, Finland). Exposed and unexposed samples were randomly placed on 96-well plates, shipped on dry ice, and analysed using high-throughput nuclear magnetic resonance metabolomics [23]. Further details of the Nightingale Health nuclear magnetic resonance biomarker platform and experiment process have been previously described [24]. Briefly, metabolites were quantified using the Nightingale Health high-throughput nuclear magnetic resonance (NMR) metabolomics platform, which provides standardized measurements of a broad panel of circulating biomarkers, including lipids, fatty acids, amino acids, and glycolysis-related metabolites. The platform quantifies both absolute concentrations and relative proportions of metabolites.

Fasting blood glucose and HbA1c were measured in venous blood using routine clinical chemistry procedures. HbA1c indicates the average level of blood glucose in the past three months.

### Metal analysis in blood

Blood samples were diluted 1:10 in an alkali buffer, sonicated for 5 min and separated by centrifugation for 10 min at 1000 rpm. Lead and cadmium concentrations were measured by inductively coupled plasma–mass spectrometry (ICP-MS; Agilent 7900, Agilent Technologies, Santa Clara, USA) equipped with a collision/reaction system in helium (for cadmium) and no gas (lead) mode [25]. The analysis accuracy was determined by analyzing certified reference material (Seronorm L1-WB 2011920 and L2-WB 2011921) together with the samples. The limit of detection (LOD) was calculated as the average element level in analysed blank samples plus three standard deviations.

### Covariates

The baseline questionnaire included information on: age (continuous), sex (male, female), education (low/medium-secondary studies or less, high-university or more), country of origin (European, other), center (Spain, Sweden, Denmark), civil status (married/cohabiting, living alone), smoking (never, former, current), alcohol use (yes, no), physical activity using the International Physical activity Questionnaire [26] which was used to generate total metabolic equivalents of task (METs, continuous), season (Spring, Summer, Fall, Winter), diet (continuous diet score ranging from 0–9 built from a modified Mediterranean Diet Adherence Screener) [27], and Pittsburgh Sleep Quality Index (continuous) [28]. Concentrations of blood cadmium and lead from blood were included (continuous).

### Statistical analysis

For descriptive purposes, the correlations between the cardiometabolic outcomes stratified by shift type were examined by Pearson correlation coefficients adjusting for age. Linear regression models (for continuous outcomes) or logistic regression models (for binary outcomes) were utilized to examine the associations between night shift exposure and outcomes. The association between night shift work and our outcomes were investigated in two modes: Model 1, adjusted for age, sex, and center; Model 2, further adjusted for civil status, smoking, alcohol use, physical activity, country of origin, and season. *A priori* model 2 was considered our primary model.

Regression models with a dummy exposure variable were used to examine associations between day shift work (reference), permanent night shift work, and rotating shift work with nights and cardiometabolic outcomes. Additional analyses were conducted stratified by sex to examine potential sex-related differences.

In dose-response analyses, outcomes that were significantly associated with shift exposure in linear models were further examined with respect to (i) number of consecutive night shifts [day shift (reference), 1–2 night shifts, ≥3 night shifts]; (ii) shifts per week [day shift (reference), 1–3 night shifts, ≥4 night shifts]; and (iii) history of working night shifts [<5 years (reference), 5–19 years, ≥20 years] with each of the cardiometabolic outcomes under examination. The Cochran-Armitage Trend test was used to estimate *P* value for trend across exposure groups.

Some sensitivity analyses were performed. (i) For the blood pressure-related outcomes, analyses were run in individuals who did not take anti-hypertensive medications. We also (ii) adjusted all models for the time of sampling (to account for variability for those who had their blood sampled at earlier/later time periods outside of the 6 a.m.–10 a.m. time window). Analyses were also run (iii) with additional adjustment for exposure to metals associated with cardiovascular disease via blood concentrations of cadmium and lead for all outcomes, (iv) adjusting for diet, (v) adjusting for sleep quality using the 21-point Pittsburgh Sleep Quality Index sleep score, (vi) restricting the day shift group (reference) to those with no prior history of night shift work, and (vii) removing the 54 night shift workers who were not working night shifts a full three years prior to enrollment.

Analyses used SAS (9.4, SAS Institute, Cary, NC) and R (3.2.2).

## Results

The study population included 860 individuals (Fig. S1, Table 1), the majority of whom were female (87%) and health care workers (96%). The sample included 58% night and 42% day shift workers (mean age 43). The night shift workers were more likely to be current (22% vs 14%) or former (31% vs. 29%) smokers and were also more likely to drink alcohol (81% vs 77%). Night shift workers had higher concentrations of cadmium in blood 0.505 µg/L (SD = 0.678) vs 0.400 µg/L (SD = 0.441) (Table 1).

**Table 1.** Characteristics of the study population

Characteristics	Night shift (*n* = 498)	Day shift (*n* = 362)	Total (*N* = 860)
Sex, %			
Female	87.3	86.2	86.9
Male	12.7	13.8	13.1
Shift schedule, %			
Permanent day	0	98.9	41.6
Permanent evening without nights	0	0.3	0.1
Permanent night	63.4	0	36.7
Rotating without nights	0	0.8	0.4
Rotating with nights	36.6	0	21.2
Educational level, %			
Low/Medium	31.7	31.5	31.6
High	68.3	68.5	68.4
Civil status, %			
Single/divorced/widow(er)	36.1	38.1	37.0
Married/cohabiting	63.9	61.9	63.0
Country of origin, %			
European	89.0	87.9	88.5
Other	11.0	12.2	11.5
Smoking, %			
Never	47.6	57.2	51.6
Former	30.7	28.5	29.8
Current	21.7	14.4	18.6
Snuff, %^a^			
Yes	21.2	16.5	18.8
No	78.8	83.5	81.2
Alcohol, %			
Yes	66.9	68.2	67.4
No	33.1	31.8	32.6
Metabolic syndrome^b^, %			
No	78.7	80.7	79.5
Yes	21.3	19.3	20.5
Age, years, mean ± SD	43.0 ± 12.0	43.8 ± 13.1	43.3 ± 12.5
Diet score, mean ± SD^c^	5.4 ± 1.5	5.3 ± 1.6	5.3 ± 1.5
Physical activity score, mean ± SD	2098 ± 2204	2180 ± 2077	2133 ± 2150
Sleep score, mean ± SD	9.2 ± 2.9	8.2 ± 2.6	8.7 ± 2.8
BMI (kg/m^2^), mean ± SD	27.1 ± 5.8	25.8 ± 4.9	26.6 ± 5.4
Waist circumference (cm), mean ± SD	88.0 ± 14.5	85.6 ± 13.2	87.0 ± 14.0
Hip circumference (cm) mean ± SD	105.0 ± 12.5	102.0 ± 12.1	103.7 ± 12.4
Waist hip ratio, mean ± SD	0.8 ± 0.1	0.8 ± 0.1	0.84 ± 0.1
Systolic Blood pressure (mmHg), mean ± SD	113.7 ± 14.9	111.0 ± 15.2	112.6 ± 15.1
Diastolic Blood pressure (mmHg), mean ± SD	76.5 ± 10.6	74.6 ± 10.5	75.7 ± 10.6
Lead (µg/L)^d^, mean ± SD	10.7 ± 5.2	11.1 ± 5.4	10.9 ± 5.3
Cadmium (µg/L)^d^, mean ± SD	0.51 ± 0.68	0.40 ± 0.44	0.45 ± 0.58

SD—standard deviation.

a Only available in Swedish sample (*n* = 170).

b Metabolic syndrome was defined according to the International Diabetes Federation: (a) central obesity, defined as waist circumference ⩾80 cm for women and ⩾94 cm for men, and (b) at least two of the following: (i) low serum HDL-Cholesterol (<1.29 mmol/l for women and <1.03 mmol/l for men), or specific therapy (medication) for that lipid abnormality; (ii) high serum triglycerides (⩾1.7 mmol/l), or specific treatment for that lipid abnormality; (iii) high blood pressure (⩾130 mm Hg systolic and/or ⩾85 mm Hg diastolic) or antihypertensive medication; and (iv) raised fasting glucose levels (⩾5.6 mmol/l) or diagnosed type two diabetes mellitus or medication.

c Available for 386.

d Available for 436.

Night shift work was associated with higher mean SBP (β = 1.89, 95%CI 0.00, 3.79 mmHg), higher mean BMI (β = 1.14, 95%CI 0.44, 1.84 kg/m^2^), higher odds of hypertension (OR = 1.38, 95%CI 1.00, 1.89) and higher odds of overweight/obesity (OR = 1.37, 95%CI 1.02, 1.82), compared to day shift workers in the full population (Table 2, Model 2). Among women, night shift work was more strongly associated with higher SBP (β = 2.74, 95% CI 0.72, 4.76 mmHg), higher BMI (β = 1.18, 95% CI 0.42, 1.94 kg/m^2^), a higher odds of hypertension (OR = 1.53, 95% CI 1.08, 2.17), and a higher odds of overweight/obesity (OR = 1.45, 95% CI 1.07, 1.99) (Table 2). None of the findings among male night shift workers reached statistical significance.

**Table 2.** Sex-stratified results from linear and logistic regression models examining associations between night shift work (permanent night or rotating night) and cardiometabolic outcomes (*N* = 860), using permanent day as a reference

	Full population (*N *= 860: 498 night, 362 day)		Men (*n *= 114: 63 night, 50 day)		Women (*n *= 746: 435 night, 312 day)	
	Estimate (95% CI)		Estimate (95% CI)		Estimate (95% CI)	
Outcome	Model 1^a^	Model 2^b^	Model 1^a^	Model 2^b^	Model 1^a^	Model 2^b^
Systolic BP (mmHg) (Beta)	1.68 (−0.20, 3.55)	1.89 (0.00, 3.79)	-3.67 (−9.12, 1.77)	-3.19 (−8.34, 1.96)	2.52 (0.54, 4.51)	2.74 (0.72, 4.76)
Diastolic BP (mmHg) (Beta)	1.00 (−0.36, 2.36)	0.94 (−0.43, 2.32)	−1.75 (−5.82, 2.33)	−1.5 (−5.34, 2.35)	1.44 (0.01, 2.88)	1.38 (−0.07, 2.82)
BMI (kg/m^2^) (Beta)	1.15 (0.45, 1.85)	1.14 (0.44, 1.84)	0.97 (−0.81, 2.74)	1.10 (−0.65, 2.85)	1.17 (0.41, 1.93)	1.18 (0.42, 1.94)
WHR (Beta)	0.01 (−0.00, 0.02)	0.01 (−0.00, 0.02)	0.01 (−0.03, 0.05)	0.02 (−0.01, 0.05)	0.01 (−0.00, 0.02)	0.01 (−0.00, 0.02)
Hypertension (OR)	1.36 (1.00, 1.84)	1.38 (1.00, 1.89)	0.76 (0.35, 1.68)	0.86 (0.33, 2.20)	1.52 (1.09, 2.13)	1.53 (1.08, 2.17)
Overweight/obese vs. normal/underweight (OR)	1.36 (1.02, 1.79)	1.37 (1.02, 1.82)	0.87 (0.39, 1.90)	0.90 (0.38, 2.15)	1.45 (1.07, 1.96)	1.45 (1.07, 1.99)
Moderate/high abdominal obesity vs normal (OR)	1.34 (0.98, 1.83)	1.32 (0.96, 1.81)	1.82 (0.80, 4.14)	2.35 (0.90, 6.12)	1.27 (0.90, 1.78)	1.24 (0.88, 1.74)

a Adjusted for age, sex, center, education level, civil status, physical activity, smoking status, alcohol consumption, country of origin, and season.

b Adjusted for age, sex, center, education level, civil status, physical activity, smoking status, alcohol consumption, country of origin, season, and diet score (modified MEDAS). No adjustment for snuff use.

In secondary analyses, where regression models with a dummy exposure variable were used to compare associations between (i) permanent night shift work and day shift work and (ii) rotating night shift work and day shift work (Table S1), we found that permanent night shift work was associated more strongly with negative cardiometabolic outcomes than rotating shift work with nights. This was most evident for SBP (β = 2.94, 95%CI 0.86, 5.02 mmHg vs. β = −0.74, 95%CI −3.69, 2.21 mmHg) for permanent nights vs. days and rotating with nights vs. days, respectively, BMI (β = 1.32, 95%CI 0.51, 2.12 vs. β = 0.83, 95%CI −0.32, 1.97) and hypertension (OR = 1.61, 95% CI 1.12, 2.30 vs. OR = 0.97, 95% CI 0.60, 1.56).

In sensitivity analyses, when we adjusted for lead and cadmium concentrations in blood (*n* = 436), we found a somewhat stronger association between night shift work and (i) SBP, (ii) DBP, and (iii) hypertension (Table S2). Results were similar when excluding antihypertensive users and adjusting for sampling time in sensitivity analyses (data not shown). For blood pressure and markers of obesity, further adjustment for diet (Table S3) and sleep score (Table S4) produced similar results. When using a reference group of those who never worked night shifts, we observed a somewhat weaker associations for SBP but stronger associations for markers of obesity (Table S5). Lastly, results were largely unchanged when we removed the 54 participants who were not working night shifts for three years prior to enrollment (Table S6).

Night shift work was also associated with some plasma metabolite concentrations and fractions (Table 3, Model 2). Fractions of fatty acids omega 3 and 6, polyunsaturated fatty acids (PUFAs), and linolenic acid of total fatty acids were negatively associated with working night shift. Similarly, both concentration and fraction of the PUFA docosahexaenoic acid (DHA) were negatively associated with night shift work. Monounsaturated and saturated fatty acid fractions of total fatty acids were positively associated with night shift work, whereas PUFAs fraction of monounsaturated fatty acids were negatively associated with night shift work. Night shift work was negatively associated with concentrations of the amino acids alanine and glycine and positively associated with total and the individual branched-chain amino acids isoleucine and valine, as well as the aromatic amino acid phenylalanine. Overall, our findings were robust to additional adjustment for diet in the subset with available information (Table S3).

**Table 3.** Associations between night shift work (permanent or rotating nights) and cardiometabolic biomarkers as obtained from linear regression models (*N* = 734) using permanent day work as the reference

	Model 1^a^	Model 2^b^
	Beta (95% CI)	Beta (95% CI)
Cholesterol (mmol/l)		
Total cholesterol	−0.06 (−0.21,0.09)	−0.08 (−0.23, 0.08)
VLDL cholesterol	0.001 (−0.03, 0.04)	−0.01 (−0.04, 0.03)
LDL cholesterol	−0.05 (−0.12, 0.02)	−0.06 (−0.13, 0.01)
HDL cholesterol	−0.01 (−0.06, 0.05)	−0.002 (−0.06, 0.05)
Total triglycerides (mmol/l)	0.02 (−0.04, 0.09)	0.01 (−0.06, 0.08)
Fatty acids (mmol/l)		
Total fatty acids	0.19 (−0.19, 0.56)	0.13 (−0.25, 0.52)
Omega-3	−0.02 (−0.04, 0.01)	−0.02 (−0.04, 0.01)
Omega-6	−0.01 (−0.14, 0.12)	−0.03 (−0.15, 0.10)
Polyunsaturated fatty acids	−0.03 (−0.17, 0.12)	−0.04 (−0.19, 0.10)
Monounsaturated fatty acids	0.11 (−0.01, 0.23)	0.09 (−0.03, 0.21)
Saturated fatty acids	0.10 (−0.03, 0.23)	0.08 (−0.05, 0.22)
Docosahexaenoic acid	−0.02 (−0.03, -0.01)	−0.01 (−0.02, -0.01)
Linoleic acid	−0.03 (−0.16, 0.09)	−0.05 (−0.18, 0.07)
Fatty acid ratios (%)		
Omega-3 fatty acids to total fatty acids	−0.23 (−0.38, -0.08)	−0.19 (−0.35, -0.04)
Omega-6 fatty acids to total fatty acids	−0.62 (−1.04, -0.20)	−0.59 (−1.02, -0.17)
Polyunsaturated fatty acids to total fatty acids	−0.85 (−1.26, -0.42)	−0.79 (−1.21, -0.36)
Monounsaturated fatty acids to total fatty acids	0.52 (0.17, 0.88)	0.46 (0.10, 0.82)
Saturated fatty acids to total fatty acids	0.32 (0.08, 0.56)	0.33 (0.09, 0.57)
Docosahexaenoic acid to total fatty acids	−0.17 (−0.24, -0.10)	−0.15 (−0.22, -0.08)
Polyunsaturated fatty acids to monounsaturated fatty acids	−0.07 (−0.12, -0.03)	−0.07 (−0.11, -0.02)
Omega-6 fatty acids to omega-3 fatty acids	0.28 (−1.15, 1.71)	−0.01 (−1.42, 1.41)
Linoleic acid to total fatty acids	−0.74 (−1.13, -0.35)	−0.75 (−1.14, -0.36)
Apolipoproteins (g/l)		
Apolipoprotein B	−0.01 (−0.04, 0.02)	−0.02 (−0.05, 0.01)
Apolipoprotein A1	0.002 (−0.04, 0.04)	0.002 (−0.04, 0.05)
Ratio of apolipoprotein B to A1 (ratio)	−0.01 (−0.03, 0.01)	−0.01 (−0.04, 0.01)
Amino acids (mmol/l)		
Alanine	−0.02 (−0.03, -0.01)	−0.02 (−0.03, -0.01)
Glycine	−0.01 (−0.02, -0.001)	−0.01 (−0.02, -0.001)
Histidine	−0.001 (−0.002, 0.001)	−0.001 (−0.002, 0.001)
Branched-chain amino acids (mmol/l)		
Total branched-chain amino acids	0.02 (0.01, 0.04)	0.02 (0.01, 0.04)
Isoleucine	0.01 (0.002, 0.01)	0.01 (0.002, 0.01)
Leucine	0.01 (−0.001, 0.01)	0.01 (−0.001, 0.01)
Valine	0.01 (0.002, 0.02)	0.01 (0.002, 0.02)
Aromatic amino acids (mmol/l)		
Phenylalanine	0.002 (0.001, 0.003)	0.002 (0.0002, 0.003)
Tyrosine	0.001 (−0.002, 0.003)	0.001 (−0.002, 0.003)
Glycolysis related metabolites (mmol/l)		
Glucose	−0.06 (−0.21, 0.10)	−0.09 (−0.24, 0.07)
Lactate	−0.05 (−0.13, 0.03)	−0.06 (−0.14, 0.02)
Fluid balance (mmol/l)		
Creatinine	−0.70 (−2.26, 0.86)	−0.68 (−2.26, 0.90)
Albumin (g/l)	0.25 (−0.64, 1.14)	0.16 (−0.73, 1.06)
Inflammation (mmol/l)		
Glycoprotein acetyls	0.01 (−0.01, 0.03)	0.01 (−0.01, 0.03)
FBG^c^	0.07 (−0.08, 0.22)	0.03 (−0.13, 0.19)
HbA1c^c^	0.08 (−0.80, 0.96)	−0.11 (−1.01, 0.79)

a Adjusted for age, sex, and center.

b Adjusted for age, sex, center, education level, civil status, physical activity, smoking status, alcohol consumption, country of origin, and season.

c Only available in Swedish sample (*n* = 156 for FBG and *n* = 169 for HbA1c).

CI—confidence interval.

Compared to day shift workers, night shift workers did not show any apparent differences in plasma concentrations of VLDL, LDL, HDL, apolipoprotein A1 respective B and their ratio, triglycerides, total fatty acid, omega 3 and 6, creatinine, albumin, glucose or glycoprotein acetyls (Table 3). For the Swedish subsample, there was no differences between night vs day shift workers in fasting blood glucose or HbA1c (*N* = 155).

There was a dose-response relationship between the number of consecutive night shifts and a higher SBP as well as a higher BMI (Table 4). There was also evidence of a dose-response relationship between greater intensity of night shifts/week and a higher BMI as well as for a higher SBP and a higher BMI based on increasing prior duration in night shift work. Among metabolites that were significantly different between night and day shift workers, dose-response relationships were found with increasing number of (i) consecutive night shifts and (ii) night shifts with greater intensity of night shifts/week and: higher fractions of omega 3 and 6 fatty acids, PUFA, monosaturated, saturated, DHA and linoleic fatty acids, concentrations of DHA, as well as amino acids valine and phenylalanine (Table 4). The clearest dose-relationships were observed for fractions of fatty acids and number of consecutive nights.

**Table 4.** Linear regression for cardiometabolic risk factors and plasma metabolites that were significantly associated with working night shift according to number of consecutive night shifts, intensity, and history

Consecutive	Beta (95% CI)^a^	Intensity	Beta (95% CI)^a^	History	Beta (95% CI),^a^
Systolic blood pressure					
Day shift		Day shift		<5 years	
1-2 night shifts	1.22 (−1.04, 3.48)	1-3 night shifts	2.42 (0.141, 4.69)	5-19 years	0.34 (−1.74, 2.42)
≥3 night shifts	2.77 (0.53, 5.02)	≥4 night shifts	1.58 (−0.76, 3.91)	≥20 years	5.11 (2.151, 8.06)
*P* for trend	0.02	*P* for trend	0.19	*P* for trend	<0.001
Continuous	1.38 (0.25, 2.51)	Continuous	0.90 (−0.28, 2.09)	Continuous	2.00 (0.30, 3.70)
Body mass index					
Day shift		Day shift		<5 years	
1-2 night shifts	0.78 (−0.09, 1.66)	1-3 night shifts	1.23 (0.35, 2.11)	5-19 years	0.57 (−0.24, 1.37)
≥3 night shifts	1.57 (0.70, 2.43)	≥4 night shifts	1.14 (0.24, 2.04)	≥20 years	1.37 (0.22, 2.52)
*P* for trend	<0.001	*P* for trend	0.01	*P* for trend	0.02
Continuous	0.78 (0.34, 1.21)	Continuous	0.64 (0.21, 1.06)	Continuous	0.64 (0.09, 1.19)
Docosahexaenoic acid					
Day shift	Reference	Day shift	Reference	<5 years	Reference
1-2 night shifts	−0.01 (−0.03, -0.003)	1-3 night shifts	−0.01 (−0.02, -0.001)	5-19 years	−0.01 (−0.03, -0.03)
≥3 night shifts	−0.01 (−0.03, -0.003)	≥4 night shifts	−0.02 (−0.03, -0.01)	≥20 years	−0.02 (−0.04, -0.002)
*P* for trend	0.01	*P* for trend	0.002	*P* for trend	0.02
Continuous	−0.004 (−0.01, -0.001)	Continuous	−0.004 (−0.01, -0.002)	Continuous	−0.001 (−0.001, -0.0001)
Omega-3 fatty acids to total fatty acids					
Day shift	Reference	Day shift	Reference	<5 years	Reference
1-2 night shifts	−0.19 (−0.38, -0.01)	Day shift	−0.20 (−0.38, -0.01)	5-19 years	−0.22 (−0.42, -0.02)
≥3 night shifts	−0.19 (−0.37, -0.002)	1-3 night shifts	−0.18 (−0.36, 0.01)	≥20 years	−0.22 (−0.51, 0.07)
*P* for trend	0.03	≥4 night shifts	0.04	*P* for trend	0.07
Continuous	−0.05 (−0.10, 0.004)	*P* for trend	−0.06 (−0.10, -0.02)	Continuous	−0.01 (−0.02, 0.002)
Omega-6 fatty acids to total fatty acids					
Day shift	Reference	Day shift	Reference	<5 years	Reference
1-2 night shifts	−0.20 (−0.72, 0.32)	Day shift	−0.66 (−1.19, -0.14)	5-19 years	−0.10 (−0.69, 0.50)
≥3 night shifts	−0.98 (−1.49, -0.47)	1-3 night shifts	−0.52 (−1.04, -0.004)	≥20 years	0.12 (−0.73, 0.97)
*P* for trend	<0.001	≥4 night shifts	0.02	*P* for trend	0.88
Continuous	−0.27 (−0.40, -0.13)	*P* for trend	−0.15 (−0.27, -0.03)	Continuous	0.01 (−0.03, 0.04)
Polyunsaturated fatty acids to total fatty acids					
Day shift	Reference	Day shift	Reference	<5 years	Reference
1-2 night shifts	−0.40 (−0.92, 0.13)	1-3 night shifts	−0.86 (−1.39, -0.33)	5-19 years	−0.31 (−0.92, 0.29)
≥3 night shifts	−1.17 (−1.68, -0.66)	≥4 night shifts	−0.70 (−1.22, -0.18)	≥20 years	−0.10 (−0.96, 0.76)
*P* for trend	<0.001	*P* for trend	0.003	*P* for trend	0.65
Continuous	−0.31 (−0.45, -0.18)	Continuous	−0.21 (−0.32, -0.09)	Continuous	−0.002 (−0.03, 0.03)
Monounsaturated fatty acids to total fatty acids					
Day shift	Reference	Day shift	Reference	<5 years	Reference
1-2 night shifts	0.15 (−0.29, 0.59)	1-3 night shifts	0.50 (0.05, 0.94)	5-19 years	0.10 (−0.40, 0.60)
≥3 night shifts	0.74 (0.30, 1.17)	≥4 night shifts	0.40 (−0.03, 0.84)	≥20 years	0.06 (−0.65, 0.77)
*P* for trend	<0.001	*P* for trend	0.04	*P* for trend	0.80
Continuous	0.22 (0.11, 0.34)	Continuous	0.12 (0.02, 0.22)	Continuous	0.003 (−0.02, 0.03)
Saturated fatty acids to total fatty acids					
Day shift	Reference	Day shift	Reference	<5 years	Reference
1-2 night shifts	0.24 (−0.05, 0.54)	1-3 night shifts	0.36 (0.07, 0.66)	5-19 years	0.21 (−0.12, 0.55)
≥3 night shifts	0.43 (0.14, 0.72)	≥4 night shifts	0.30 (0.01, 0.59)	≥20 years	0.04 (−0.44, 0.51)
*P* for trend	0.003	*P* for trend	0.02	*P* for trend	0.66
Continuous	0.09 (0.01, 0.17)	Continuous	0.09 (0.02, 0.15)	Continuous	−0.001 (−0.02, 0.02)
Docosahexaenoic acid to total fatty acids					
Day shift	Reference	Day shift	Reference	<5 years	Reference
1-2 night shifts	−0.11 (−0.19, -0.02)	1-3 night shifts	−0.13 (−0.22, -0.05)	5-19 years	−0.08 (−0.17, 0.02)
≥3 night shifts	−0.18 (−0.27, -0.10)	≥4 night shifts	−0.15 (−0.24, -0.07)	≥20 years	−0.11 (−0.24, 0.02)
*P* for trend	<0.001	*P* for trend	<0.001	*P* for trend	0.08
Continuous	−0.05 (−0.07, -0.02)	Continuous	−0.04 (−0.06, -0.02)	Continuous	−0.01 (−0.01, -0.00004)
Polyunsaturated fatty acids to monounsaturated fatty acids					
Day shift	Reference	Day shift	Reference	<5 years	Reference
1-2 night shifts	−0.03 (−0.08, 0.03)	1-3 night shifts	−0.07 (−0.13, -0.02)	5-19 years	−0.02 (−0.08, 0.05)
≥3 night shifts	−0.10 (−0.16, -0.05)	≥4 night shifts	−0.06 (−0.11, -0.003)	≥20 years	−0.004 (−0.09, 0.08)
*P* for trend	<0.001	*P* for trend	0.02	*P* for trend	0.84
Continuous	−0.02 (−0.04, -0.02)	Continuous	−0.02 (−0.03, -0.01)	Continuous	−0.0003 (−0.004, 0.003)
Linoleic acid to total fatty acids					
Day shift	Reference	Day shift	Reference	<5 years	Reference
1-2 night shifts	−0.66 (−1.14, -0.18)	1-3 night shifts	−0.76 (−1.25, -0.28)	5-19 years	−0.23 (−0.77, 0.31)
≥3 night shifts	−0.88 (−1.35, -0.40)	≥4 night shifts	−0.79 (−1.27, -0.32)	≥20 years	−0.10 (−0.88, 0.67)
*P* for trend	<0.001	*P* for trend	<0.001	*P* for trend	0.67
Continuous	−0.25 (−0.38, -0.12)	Continuous	−0.19 (−0.30, -0.09)	Continuous	−0.001 (−0.03, 0.03)
Alanine					
Day shift	Reference	Day shift	Reference	<5 years	Reference
1-2 night shifts	−0.03 (−0.05, -0.02)	1-3 night shifts	−0.02 (−0.04, -0.01)	5-19 years	−0.02 (−0.04, -0.01)
≥3 night shifts	−0.01 (−0.03, 0.004)	≥4 night shifts	−0.02 (−0.04, -0.01)	≥20 years	−0.03 (−0.05, -0.01)
*P* for trend	0.04	*P* for trend	0.003	*P* for trend	0.01
Continuous	−0.003 (−0.01, 0.001)	Continuous	−0.01 (−0.01, -0.002)	Continuous	−0.001 (−0.002, -0.0003)
Glycine					
Day shift	Reference	Day shift	Reference	<5 years	Reference
1-2 night shifts	−0.01 (−0.03, -0.003)	1-3 night shifts	−0.01 (−0.02, 0.004)	5-19 years	−0.01 (−0.02, 0.01)
≥3 night shifts	−0.01 (−0.02, 0.01)	≥4 night shifts	−0.01 (−0.03, -0.002)	≥20 years	−0.03 (−0.05, -0.01)
*P* for trend	0.14	*P* for trend	0.02	*P* for trend	0.01
Continuous	−0.003 (−0.01, 0.001)	Continuous	−0.002 (−0.005, 0.001)	Continuous	−0.001 (−0.002, -0.001)
Total branched-chain amino acids					
Day shift	Reference	Day shift	Reference	<5 years	Reference
1-2 night shifts	0.02 (−0.001, 0.05)	1-3 night shifts	0.02 (−0.001, 0.05)	5-19 years	−0.01 (−0.04, 0.02)
≥3 night shifts	0.03 (0.002, 0.05)	≥4 night shifts	0.02 (0.001, 0.05)	≥20 years	−0.01 (−0.05, 0.03)
*P* for trend	0.02	*P* for trend	0.03	*P* for trend	0.54
Continuous	0.01 (0.003, 0.02)	Continuous	0.01 (0.0004, 0.01)	Continuous	−0.0003 (−0.001, 0.001)
Isoleucine					
Day shift	Reference	Day shift	Reference	<5 years	Reference
1-2 night shifts	0.01 (0.002, 0.01)	1-3 night shifts	0.01 (0.001, 0.01)	5-19 years	0.0002 (−0.01, 0.01)
≥3 night shifts	0.01 (0.001, 0.01)	≥4 night shifts	0.01 (0.002, 0.01)	≥20 years	0.0004 (−0.01, 0.01)
*P* for trend	0.005	*P* for trend	0.002	*P* for trend	0.92
Continuous	0.002 (0.001, 0.004)	Continuous	0.002 (0.001, 0.003)	Continuous	−0.0003 (−0.002, 0.001)
Valine					
Day shift	Reference	Day shift	Reference	<5 years	Reference
1-2 night shifts	0.01 (−0.00, 0.02)	1-3 night shifts	0.01 (−0.001, 0.02)	5-19 years	−0.01 (−0.02, 0.01)
≥3 night shifts	0.01 (0.002, 0.03)	≥4 night shifts	0.01 (0.0002, 0.02)	≥20 years	−0.01 (−0.03, 0.01)
*P* for trend	0.003	*P* for trend	0.03	*P* for trend	0.41
Continuous	0.004 (0.002, 0.01)	Continuous	0.002 (0.0002, 0.01)	Continuous	−0.0002 (−0.001, 0.001)
Phenylalanine					
Day shift	Reference	Day shift	Reference	<5 years	Reference
1-2 night shifts	0.002 (−0.001, 0.004)	1-3 night shifts	0.002 (−0.0001, 0.004)	5-19 years	0.001 (−0.002, 0.003)
≥3 night shifts	0.002 (0.0004, 0.005)	≥4 night shifts	0.002 (−0.0005, 0.004)	≥20 years	−0.001 (−0.004, 0.003)
*P* for trend	0.02	*P* for trend	0.07	*P* for trend	0.87
Continuous	0.001 (0.0002, 0.001)	Continuous	0.00004 (−0.0001, 0.001)	Continuous	−0.00004 (−0.0002, 0.0001)

a Adjusted for age, sex, center, education level, civil status, physical activity, smoking status, alcohol consumption, country of origin, and season.

Some plasma metabolites that differed significantly between night shift and day shift workers were in turn associated with BMI, waist-hip ratio, SBP and DBP and hypertension, and the associations were in general stronger in the night shift group (Fig. S2, adjusted for age; Table S7). Fraction of omega 3 was negatively associated with BMI and DBP (β = −0.82, 95%CI −1.33 to −0.3 and β = −1.41, 95%CI −2.34 to −0.47, respectively) (Table S7). Fractions of PUFAs were negatively associated with systolic and DBP (β = −0.71, 95%CI −1.15 to −0.28, respectively β = −0.76, 95%CI −1.07 to −0.44,). Fractions of monounsaturated fatty acids were positively associated with hypertension (OR = 1.17, 95%CI 1.02–1.34) and fractions of saturated fatty acids (OR = 1.47, 95%CI 1.05–2.05). The amino acid alanine was significantly positively associated with overweight and abdominal obesity in the night shift group, but the confidence intervals were very wide (OR = 16.70, 95%CI 1.16–240.49, respectively OR = 37.43, 95%CI 1.30–999.32).

## Discussion

In our study, we demonstrated that night shift work was associated with elevated cardiometabolic risk factors, and we found effects for both acute (metabolic biomarkers) and chronic (BMI; whereas blood pressure is a combination of acute and chronic effects) cardiometabolic parameters. Associations were stronger among women and those with more frequent or consecutive night shifts. We also found that permanent night shift work was associated with higher cardiometabolic risks than rotating shifts including nights. Further, night shift work was associated with alterations of the plasma metabolites including fatty acids and amino acids out of which several metabolites were associated with cardiometabolic risk factors, supporting the role of metabolomic changes as mechanisms. When we explored possible dose-response relationships between night shift work and cardiometabolic risk factors and metabolite levels, there was evidence of a dose-response with consecutive night shifts and higher levels of all outcomes (exception glycine). There was also evidence of a dose-response with intensity of night shifts and higher levels of all outcomes (exception SBP).

Prior studies have reported similar findings in terms of higher BMI and abdominal obesity among night shift workers [29, 30]. In addition, prior studies show that night shift workers have a higher risk (1.31 times based on meta-analysis from 2017) of developing hypertension [31]. Our finding for hypertension was similar in magnitude (1.38 times) in the total population.

Importantly, we found that cardiometabolic risks were stronger among the female portion of the population and were also stronger among permanent night shift workers than rotating night shift workers. The stronger associations among female night shift workers may be explained by research finding that women have shorter intrinsic circadian periods and differences in sleep timing and duration than men [32]. Prior work has also suggested that men may have better shift work tolerance, although findings are inconsistent [33]. Regarding stronger associations among permanent night shift workers, a recent systematic review and meta-analysis also found that permanent night shift work was associated more strongly with overweight (RR 1.38, 95%CI 1.06, 1.80) than rotating shift work (RR 1.21, 95%CI 1.02, 1.43) [28] and that permanent and consecutive night shift strengthen the association between night shift work and hypertension [11]. We also evaluated co-exposures to metals that have been linked to circadian disruption and CVD [17, 18]. Particularly lead exposure is a known risk factor for hypertension [34], and taking co-exposure to cadmium and lead into account, strengthened the associations somewhat for night shift and blood pressure.

Our results support previous studies indicating that night shift work impacts lipid metabolism in an unhealthy direction, with a lower ratio of polyunsaturated fat and elevated ratio of saturated fat [35, 36]. One study found elevated concentrations of the branched amino acid isoleucine and leucine among night shift workers, which is in alignment with our findings [37].

Blood metabolites correlated with cardiometabolic outcomes, most strongly in night shift workers. Similar to our study, lower levels of PUFAs and higher levels of branch-chained amino acids in blood have been associated with obesity [38]. An increase in total fatty acids and saturated fatty acids has been positively associated with blood pressure, whereas an increase in PUFAs has been negatively associated with blood pressure [39]. The role of amino acids in hypertension has shown conflicting results, but one study suggested a consistent positive association between branched-chain amino acids and blood pressure [40].

Limitations include the cross-sectional design, so we cannot establish temporality between night work exposure and the investigated metabolic and cardiometabolic risk markers. While we tried to adhere to the same protocol in each center, some differences were unavoidable and therefore there could be differences by center including variations in the number of prior years of night shift work, variations of work load before the data collection, as well as variations in the timing of data collection, although when we adjusted for timing of sample collection, results remained the same as in primary analyses. In addition, there is the possibility that healthy worker bias may influence our findings, as it is more likely that those who stay in night shifts for long periods of time are those who tolerate night shift schedules well and are healthier, while those found to have a high cardiovascular risk may be advised to stop working nights. This could result in a healthier population of night shift workers than expected, and the inability to detect strong differences between night shift work and cardiometabolic outcomes.

There are also important strengths to consider including the availability of extensive data, which allowed us to include adjustment for many confounders, which were well-defined. The detailed information on shift exposure allowed us to examine potential dose-response relationships. In addition, the outcome data—including more traditional markers as well as more explorative markers (e.g. fatty acids and amino acids), as well as other occupational exposures (e.g. metals) together in one analysis, extends the work of existing studies. Furthermore, we have participants included from multiple European countries, improving the generalizability of our findings.

In this large multi-country study, night shift work was associated with higher BMI, blood pressure, and altered fatty acid profiles, with stronger associations among workers with more intensive or permanent schedules. These findings suggest biological evidence linking circadian disruption to cardiometabolic risk and highlight that work scheduling is of relevance for prevention in occupational health practice. This is important information for workplaces and the EU legislation regarding the organization of night shift work to minimize health risks.

## Supplementary Material

ckag101_Supplementary_Data

## Data Availability

Data are available upon reasonable request. Access to study data is possible through a completed data request form, available at https://www.isglobal.org/en/-/ephor. The request form is also included in online supplemental appendix 8. Participants are required to adhere to the specified collaboration requirements, namely include 2 participants per center where data are used from, no overlapping topics, relevant human subjects review and review by the steering committee. Keypoints We identified distinct metabolic alterations among night shift workers These metabolic alterations were linked to higher BMI and blood pressure, suggesting potential pathways through which circadian disruption impacts cardiometabolic health Several outcomes showed dose response associations with increasing night shift work exposure These findings highlight that work scheduling is of relevance for prevention in occupational health practice
