# Supplementary material for: Metabolic biomarkers and cardiometabolic risk among night shift workers: evidence from night shift workers in Europe
Source: Eur J Public Health. 2026 Jul 9;36(4):ckag101. doi: 10.1093/eurpub/ckag101 (PMC13348705; doi:10.1093/eurpub/ckag101)
Supplement: ckag101_Supplementary_Data [file ckag101_supplementary_data.zip › ejph-2026-01-om-0036-File002.docx]

Supplemental Table 1: Estimated risk for selected cardiovascular risk factors (N=859) **^a^** by type of night work (permanent nights vs. rotating) with permanent day as a reference, as obtained from linear (Beta) and logistic (odds ratio, OR) regression models.

| **Outcome** | **Estimate (95% CI)^b^** | |
| --- | --- | --- |
|  | Permanent night | Rotating with nights |
| Systolic BP (Beta) | 2.94 (0.86, 5.02) | -0.74 (-3.69, 2.21) |
| Diastolic BP (Beta) | 1.54 (0.02, 3.06) | -0.58 (-2.74, 1.58) |
| BMI (Beta) | 1.32 (0.51, 2.12) | 0.83 (-0.32, 1.97) |
| WHR (Beta) | 0.01 (0.00, 0.02) | 0.00 (-0.01, 0.02) |
| Hypertension (OR) | 1.61 (1.12, 2.30) | 0.97 (0.60, 1.56) |
| Overweight/obese vs. normal/underweight (OR) | 1.41 (1.02, 1.95) | 1.43 (0.91, 2.25) |
| Moderate/high abdominal obesity vs normal (OR) | 1.38 (0.96, 1.99) | 1.23 (0.76, 1.98) |
| ^a^ 359 working permanent day, 315 permanent night, 185 rotating with nights)  ^b^ Adjusted for age, sex, center, education level, civil status, physical activity, smoking status, alcohol consumption, country of origin, and season | | |
