# Supplementary material for: Metabolic biomarkers and cardiometabolic risk among night shift workers: evidence from night shift workers in Europe
Source: Eur J Public Health. 2026 Jul 9;36(4):ckag101. doi: 10.1093/eurpub/ckag101 (PMC13348705; doi:10.1093/eurpub/ckag101)
Supplement: ckag101_Supplementary_Data [file ckag101_supplementary_data.zip › ejph-2026-01-om-0036-File003.docx]

Supplemental Table 2: Estimated risk for selected cardiovascular risk factors for night shift work (permanent or rotating) with permanent day as a reference, as obtained from linear (Beta) and logistic (odds ratio, OR) regression models. Results are shown for full population (N=860), and population with data on metal concentrations in blood (n=436) without and with adjustment for concentrations of lead and cadmium.

| **Outcome** | **Primary model among full population (N=860),** estimate (95% CI) | **Population with data on metals (n=436),** estimate (95% CI) | |
| --- | --- | --- | --- |
|  |  | Primary model^a^ | With adjustment for lead and cadmium^b^ |
| Systolic BP (Beta) | 1.89 (0.00, 3.79) | 4.13 (1.16, 7.09) | 4.27 (1.31, 7.22) |
| Diastolic BP (Beta) | 0.94 (-0.43, 2.32) | 1.54 (-0.50, 3.58) | 1.63 (-0.40, 3.66) |
| BMI (Beta) | 1.14 (0.44, 1.84) | 0.83 (-0.13, 1.80) | 0.79 (-0.17, 1.74) |
| WHR (Beta) | 0.01 (-0.00, 0.02) | 0.01 (-0.01, 0.02) | 0.01 (-0.01, 0.02) |
| Hypertension (OR) | 1.38 (1.00, 1.89) | 1.67 (1.08, 2.59) | 1.70 (1.10, 2.63) |
| Overweight/obese vs. normal/underweight (OR) | 1.37 (1.02, 1.82) | 1.15 (0.77, 1.71) | 1.14 (0.76, 1.70) |
| Moderate/high abdominal obesity vs normal (OR) | 1.32 (0.96, 1.81) | 1.20 (0.77, 1.87) | 1.15 (0.74, 1.80) |
| ^a^ Adjusted for age, sex, center, education level, civil status, physical activity, smoking status, alcohol consumption, country of origin, and season  ^b^ Adjusted for age, sex, center, education level, civil status, physical activity, smoking status, alcohol consumption, country of origin, season, lead, cadmium | | | |
