# Supplementary material for: Metabolic biomarkers and cardiometabolic risk among night shift workers: evidence from night shift workers in Europe
Source: Eur J Public Health. 2026 Jul 9;36(4):ckag101. doi: 10.1093/eurpub/ckag101 (PMC13348705; doi:10.1093/eurpub/ckag101)
Supplement: ckag101_Supplementary_Data [file ckag101_supplementary_data.zip › ejph-2026-01-om-0036-File004.docx]

Supplement Table 3. Associations between night shift work (permanent or rotating nights) and cardiometabolic biomarkers with additional adjustment for diet (n=368 for blood pressure and obesity markers, n=369 for metabolomic markers).

|  | Model 2^a^ | Model 3^b^ |
| --- | --- | --- |
|  | Beta (95% CI), *p-value* | Beta (95% CI), *p-value* |
| Systolic BP (mmHg) (Beta) | 2.25 (-0.49, 4.99) | 2.20 (-0.53, 4.94) |
| Diastolic BP (mmHg) (Beta) | 0.86 (-1.29, 3.00) | 0.83 (-1.32, 2.98) |
| BMI (kg/m^2^) (Beta) | 1.10 (0.14, 2.07) | 1.12 (0.16, 2.08) |
| WHR (Beta) | 0.01 (-0.00, 0.03) | 0.01 (-0.00, 0.03) |
| Hypertension (OR) | 1.44 (0.87, 2.40) | 1.44 (0.86, 2.39) |
| Overweight/obese vs. normal/underweight (OR) | 1.32 (0.86, 2.03) | 1.33 (0.86, 2.04) |
| Moderate/high abdominal obesity vs normal (OR) | 2.11 (1.25, 3.58) | 2.15 (1.26, 3.68) |
| Cholesterol (mmol/l) |  |  |
| Total cholesterol | -0.03 (-0.28, 0.23) | -0.03 (-0.28, 0.22) |
| VLDL cholesterol | -0.01 (-0.05, 0.05) | -0.01 (-0.05, 0.04) |
| LDL cholesterol | -0.03 (-0.14, 0.08) | -0.03 (-0.15, 0.08) |
| HDL cholesterol | 0.01 (-0.07, 0.10) | 0.01 (-0.08, 0.10) |
| Total triglycerides (mmol/l) | -0.003 (-0.09, 0.09) | -0.002 (-0.09, 0.09) |
| Fatty acids (mmol/l) |  |  |
| Total fatty acids | 0.19 (-0.42, 0.80) | 0.18 (-0.43, 0.80) |
| Omega-3 | -0.01 (-0.05, 0.02) | -0.01 (-0.05, 0.02) |
| Omega-6 | -0.01 (-0.23, 0.20) | -0.02 (-0.23, 0.20) |
| Polyunsaturated fatty acids | -0.02 (-0.27, 0.22) | -0.03 (-0.27, 0.21) |
| Monounsaturated fatty acids | 0.12 (-0.06, 0.30) | 0.12 (-0.07, 0.30) |
| Saturated fatty acids | 0.10 (-0.11, 0.30) | 0.09 (-0.11, 0.30) |
| Docosahexaenoic acid | -0.01 (-0.02, 0.01) | -0.01 (-0.03, 0.004) |
| Linoleic acid | -0.04 (-0.25, 0.17) | -0.04 (-0.25, 0.17) |
| Fatty acid ratios (%) |  |  |
| Omega-3 fatty acids to total fatty acids | -0.21 (-0.44, 0.01) | -0.22 (-0.44, 0.001) |
| Omega-6 fatty acids to total fatty acids | -0.77 (-1.35, -0.19) | -0.77 (-1.34, -0.18) |
| Polyunsaturated fatty acids to total fatty acids | -0.98 (-1.56, -0.40) | -0.99 (-1.57, -0.41) |
| Monounsaturated fatty acids to total fatty acids | 0.66 (0.14, 1.17) | 0.66 (0.14, 1.17) |
| Saturated fatty acids to total fatty acids | 0.32 (-0.01, 0.65) | 0.33 (0.002, 0.66) |
| Docosahexaenoic acid to total fatty acids | -0.13 (-0.23, -0.02) | -0.13 (-0.23, -0.03) |
| Polyunsaturated fatty acids to monounsaturated fatty acids | -0.10 (-0.16, -0.03) | -0.10 (-0.16, -0.03) |
| Omega-6 fatty acids to omega-3 fatty acids | -0.77 (-3.45, 1.90) | -0.71 (-3.39, 1.96) |
| Linoleic acid to total fatty acids | -0.82 (-1.36, -0.27) | -0.83 (-1.37, -0.29) |
| Apolipoproteins (g/l) |  |  |
| Apolipoprotein B | -0.01 (-0.05, 0.03) | -0.01 (-0.05, 0.03) |
| Apolipoprotein A1 | 0.02 (-0.06, 0.09) | 0.01 (-0.06, 0.09) |
| Ratio of apolipoprotein B to A1 (ratio) | -0.02 (-0.05, 0.02) | -0.01 (-0.05, 0.02) |
| Amino acids (mmol/l) |  |  |
| Alanine | -0.04 (-0.06, -0.02) | -0.04 (-0.06, -0.02) |
| Glycine | -0.01 (-0.03, 0.0001) | -0.01 (-0.03, -0.0001) |
| Histidine | -0.001 (-0.004, 0.002) | -0.001 (-0.004, 0.002) |
| Branched-chain amino acids (mmol/l) |  |  |
| Total branched-chain amino acids^a^ | 0.04 (0.004, 0.07) | 0.03 (0.003, 0.07) |
| Isoleucine | 0.01 (0.003, 0.02) | 0.01 (0.003, 0.02) |
| Leucine | 0.01 (-0.001, 0.02) | 0.01 (-0.001, 0.02) |
| Valine | 0.02 (0.0004, 0.03) | 0.02 (-0.0001, 0.03) |
| Aromatic amino acids (mmol/l) |  |  |
| Phenylalanine | 0.002 (-0.001, 0.01) | 0.002 (-0.001, 0.01) |
| Tyrosine | 0.001 (-0.003, 0.01) | 0.001 (-0.003, 0.01) |
| Glycolysis related metabolites (mmol/l) |  |  |
| Glucose | -0.05 (-0.33, 0.22) | -0.05 (-0.33, 0.22) |
| Lactate | -0.17 (-0.28, -0.05) | -0.17 (-0.29, -0.05) |
| Fluid balance (mmol/l) |  |  |
| Creatinine | 0.29 (-2.30, 2.89) | 0.24 (-2.35, 2.84) |
| Albumin (g/l) | 0.51 (-1.06, 2.08) | 0.47 (-1.09, 2.04) |
| Inflammation (mmol/l) |  |  |
| Glycoprotein acetyls | 0.01 (-0.03, 0.04) | 0.01 (-0.03, 0.04) |
| FBG^c^ | -0.08 (-0.72, 0.56) | -0.04 (-0.70, 0.61) |
| HbA1c^c^ | 1.86 (-0.61, 4.33) | 1.90 (-0.66, 4.46) |
| ^a^  Adjusted for age, sex, center, education level, civil status, physical activity, smoking status, alcohol consumption, country of origin, and season.  ^b^ Adjusted for age, sex, center, education level, civil status, physical activity, smoking status, alcohol consumption, country of origin, season, and diet (modified MEDAS).  ^c^ Only available in Swedish sample (n=27 for FBG and n=33 for HbA1c).  CI – confidence interval. | | |
