# Supplementary material for: Metabolic biomarkers and cardiometabolic risk among night shift workers: evidence from night shift workers in Europe
Source: Eur J Public Health. 2026 Jul 9;36(4):ckag101. doi: 10.1093/eurpub/ckag101 (PMC13348705; doi:10.1093/eurpub/ckag101)
Supplement: ckag101_Supplementary_Data [file ckag101_supplementary_data.zip › ejph-2026-01-om-0036-File005.docx]

Supplemental Table 4: Association between night shift work and fasting blood glucose (FBG), hemaglobinaA1c (HbA1c), and cardiometabolic biomarkers adjusted for sleep score.

| Outcome | Model 2^a^  (All) | Model 2^a^  (without missing  sleep score data) | Model 3^b^  (Adjusted for sleep score) |
| --- | --- | --- | --- |
|  | **Beta (95% CI), *p-value*** | **Beta (95% CI), *p-value*** | **Beta (95% CI), *p-value*** |
| Systolic BP (mmHg) (Beta) | 1.89 (0.00, 3.79), 0.05 | 1.98 (0.05, 3.90), 0.04 | 1.57 (-0.43, 3.57), 0.12 |
| Diastolic BP (mmHg) (Beta) | 0.94 (-0.43, 2.32), 0.18 | 0.88 (-0.53, 2.30), 0.22 | 0.47 (-0.99, 1.93), 0.53 |
| BMI (kg/m^2^) (Beta) | 1.14 (0.44, 1.84), <0.01 | 1.29 (0.56, 2.02), <0.01 | 1.13 (0.38, 1.88), <0.01 |
| WHR (Beta) | 0.01 (-0.00, 0.02), 0.26 | 0.00 (-0.01, 0.01), 0.40 | 0.00 (-0.01, 0.01), 0.52 |
| Hypertension (OR) | 1.38 (1.00, 1.89), 0.05 | 1.35 (0.97, 1.88), 0.08 | 1.28 (0.91, 1.80), 0.15 |
| Overweight/obese vs. normal/underweight (OR) | 1.37 (1.02, 1.82), 0.03 | 1.44 (1.07, 1.94), 0.02 | 1.40 (1.03, 1.90), 0.03 |
| Moderate/high abdominal obesity vs normal (OR) | 1.32 (0.96, 1.81), 0.09 | 1.25 (0.90, 1.74), 0.18 | 1.26 (0.90, 1.77), 0.17 |
| FBG^d,e^ | 0.03 (-0.13, 0.19), 0.70 | 0.01 (-0.16, 0.18), 0.87 | 0.02 (-0.15, 0.19), 0.85 |
| HbA1c^d,e^ | -0.11 (-1.01, 0.79), 0.80 | -0.11 (-1.04, 0.82), 0.81 | -0.10 (-1.03, 0.84), 0.84 |
| Cholesterol (mmol/l)^d^ |  |  |  |
| Total cholesterol | -0.08 (-0.23, 0.08), 0.33 | -0.05 (-0.25, 0.14), 0.60 | -0.07 (-0.27, 0.12), 0.47 |
| VLDL cholesterol | -0.01 (-0.04, 0.03). 0.77 | -0.03 (-0.07, 0.01). 0.21 | -0.03 (-0.08, 0.01). 0.11 |
| LDL cholesterol | -0.06 (-0.13, 0.01), 0.11 | -0.06 (-0.15, 0.03), 0.19 | -0.07 (-0.16, 0.02), 0.12 |
| HDL cholesterol | -0.002 (-0.06, 0.05), 0.93 | 0.03 (-0.03, 0.10), 0.31 | 0.04 (-0.03, 0.10), 0.28 |
| Total triglycerides (mmol/l) ^d^ | 0.01 (-0.06, 0.08), 0.75 | -0.04 (-0.12, 0.04), 0.32 | -0.05 (-0.14, 0.03), 0.20 |
| Fatty acids (mmol/l) ^d^ |  |  |  |
| Total fatty acids | 0.13 (-0.25, 0.52), 0.49 | 0.06 (-0.41, 0.54), 0.80 | -0.02 (-0.50, 0.47), 0.95 |
| Omega-3 | -0.02 (-0.04, 0.01), 0.20 | -0.02 (-0.04, 0.01), 0.26 | -0.02 (-0.05, 0.01), 0.26 |
| Omega-6 | -0.03 (-0.15, 0.10), 0.69 | -0.02 (-0.18, 0.14), 0.83 | -0.04 (-0.21, 0.12), 0.62 |
| Polyunsaturated fatty acids | -0.04 (-0.19, 0.10), 0.57 | -0.03 (-0.22, 0.15), 0.71 | -0.06 (-0.24, 0.13), 0.54 |
| Monounsaturated fatty acids | 0.09 (-0.03, 0.21), 0.14 | 0.04 (-0.11, 0.19), 0.59 | 0.02 (-0.14, 0.17), 0.84 |
| Saturated fatty acids | 0.08 (-0.05, 0.22), 0.23 | 0.06 (-0.11, 0.22), 0.51 | 0.03 (-0.14, 0.20), 0.75 |
| Docosahexaenoic acid | -0.01 (-0.02, -0.01), <0.001 | -0.01 (-0.02, -0.001), 0.03 | -0.01 (-0.02, 0.0003), 0.06 |
| Linoleic acid | -0.05 (-0.18, 0.07), 0.41 | -0.04 (-0.19, 0.12), 0.62 | -0.06 (-0.22, 0.10), 0.46 |
| Fatty acid ratios (%) ^d^ |  |  |  |
| Omega-3 fatty acids to total fatty acids | -0.19 (-0.35, -0.04), 0.01 | -0.16 (-0.34, 0.02), 0.08 | -0.13 (-0.31, 0.05), 0.16 |
| Omega-6 fatty acids to total fatty acids | -0.59 (-1.02, -0.17), 0.006 | -0.35 (-0.86, 0.16), 0.18 | -0.32 (-0.84, 0.21), 0.23 |
| Polyunsaturated fatty acids to total fatty acids | -0.79 (-1.21, -0.36), <0.001 | -0.51 (-1.03, 0.01), 0.05 | -0.45 (-0.98, 0.08), 0.10 |
| Monounsaturated fatty acids to total fatty acids | 0.46 (0.10, 0.82), 0.01 | 0.22 (-0.21, 0.66), 0.32 | 0.17 (-0.27, 0.62), 0.44 |
| Saturated fatty acids to total fatty acids | 0.33 (0.09, 0.57), 0.008 | 0.29 (0.01, 0.56), 0.04 | 0.28 (-0.01, 0.56), 0.05 |
| Docosahexaenoic acid to total fatty acids | -0.15 (-0.22, -0.08), <0.001 | -0.11 (-0.20, -0.03), 0.01 | -0.09 (-0.18, -0.01), 0.03 |
| Polyunsaturated fatty acids to monounsaturated fatty acids | -0.07 (-0.11, -0.02), 0.004 | -0.04 (-0.09, 0.02), 0.19 | -0.03 (-0.09, 0.03), 0.29 |
| Omega-6 fatty acids to omega-3 fatty acids | -0.01 (-1.42, 1.41), 0.99 | 0.84 (-0.05, 1.72), 0.06 | 0.68 (-0.22, 1.58), 0.14 |
| Linoleic acid to total fatty acids | -0.75 (-1.14, -0.36), <0.001 | -0.46 (-0.93, 0.003), 0.05 | -0.45 (-0.92, 0.03), 0.06 |
| Apolipoproteins (g/l) ^d^ |  |  |  |
| Apolipoprotein B | -0.02 (-0.05, 0.01), 0.27 | -0.02 (-0.06, 0.01), 0.23 | -0.03 (-0.06, 0.01), 0.13 |
| Apolipoprotein A1 | 0.002 (-0.04, 0.05), 0.89 | 0.02 (-0.03, 0.07), 0.44 | 0.02 (-0.03, 0.08), 0.46 |
| Ratio of apolipoprotein B to A1 (ratio) | -0.01 (-0.04, 0.01), 0.25 | -0.02 (-0.05, 0.003), 0.08 | -0.03 (-0.06, -0.001), 0.04 |
| Amino acids (mmol/l) ^d^ |  |  |  |
| Alanine | -0.02 (-0.03, -0.01), <0.001 | -0.03 (-0.04, -0.01), <0.001 | -0.03 (-0.04, -0.01), <0.001 |
| Glycine | -0.01 (-0.02, -0.001), 0.04 | -0.002 (-0.01, 0.01), 0.72 | -0.002 (-0.01, 0.01), 0.74 |
| Histidine | -0.001 (-0.002, 0.001), 0.24 | -0.001 (-0.003, 0.001), 0.20 | -0.002 (-0.004, 0.001), 0.17 |
| Branched-chain amino acids (mmol/l) ^d^ |  |  |  |
| Total branched-chain amino acids^a^ | 0.02 (0.01, 0.04), 0.02 | 0.02 (-0.003, 0.04), 0.09 | 0.02 (-0.01, 0.04), 0.15 |
| Isoleucine | 0.01 (0.002, 0.01), 0.002 | 0.01 (0.001, 0.01), 0.01 | 0.01 (0.0003, 0.01), 0.04 |
| Leucine | 0.01 (-0.001, 0.01), 0.07 | 0.004 (-0.003, 0.01), 0.29 | 0.003 (-0.004, 0.01), 0.40 |
| Valine | 0.01 (0.002, 0.02), 0.02 | 0.01 (-0.001, 0.02), 0.08 | 0.01 (-0.003, 0.02), 0.13 |
| Aromatic amino acids (mmol/l)^d^ |  |  |  |
| Phenylalanine | 0.002 (0.0002, 0.003), 0.03 | 0.002 (0.00001, 0.004), 0.05 | 0.002 (-0.0003, 0.004), 0.09 |
| Tyrosine | 0.001 (-0.002, 0.003), 0.78 | 0.001 (-0.003, 0.004), 0.65 | -0.0004 (-0.004, 0.003), 0.81 |
| Glycolysis related metabolites (mmol/l) ^d^ |  |  |  |
| Glucose | -0.09 (-0.24, 0.07), 0.26 | -0.08 (-0.27, 0.11), 0.42 | -0.10 (-0.29, 0.09), 0.31 |
| Lactate | -0.06 (-0.14, 0.02), 0.12 | -0.10 (-0.19, 0.0001), 0.05 | -0.10 (-0.19, 0.002), 0.06 |
| Fluid balance (mmol/l) ^d^ |  |  |  |
| Creatinine | -0.68 (-2.26, 0.90), 0.40 | -0.37 (-2.34, 1.60), 0.71 | -0.38 (-2.39, 1.63), 0.71 |
| Albumin (g/l) | 0.16 (-0.73, 1.06), 0.72 | 0.20 (-0.95, 1.34), 0.74 | 0.17 (-1.01, 1.34), 0.78 |
| Inflammation (mmol/l) ^d^ |  |  |  |
| Glycoprotein acetyls | 0.01 (-0.01, 0.03), 0.56 | -0.001 (-0.03, 0.03), 0.93 | -0.01 (-0.03, 0.02), 0.70 |
| ^a^ Adjusted for age, sex, center, education level, civil status, physical activity, smoking status, alcohol consumption, country of origin, and season.  ^b^ Adjusted for age, sex, center, education level, civil status, physical activity, smoking status, alcohol consumption, country of origin, season, and sleep score.  ^d^ Dayshift is the reference.  ^e^ Only in available in Sweden.  CI – confidence interval. | | | |
