# Supplementary material for: Metabolic biomarkers and cardiometabolic risk among night shift workers: evidence from night shift workers in Europe
Source: Eur J Public Health. 2026 Jul 9;36(4):ckag101. doi: 10.1093/eurpub/ckag101 (PMC13348705; doi:10.1093/eurpub/ckag101)
Supplement: ckag101_Supplementary_Data [file ckag101_supplementary_data.zip › ejph-2026-01-om-0036-File006.docx]

Supplemental Table 5. Association between night shift work and blood pressure and markers of obesity. Results are shown for a subset of the population (n=673) using never working shifts or never working night shift as the reference (n=175).

|  | Full cohort  Primary Model^a^ (n=860) | New reference (never working night shifts)  Primary Model^a^ (n=673) |
| --- | --- | --- |
|  | **Beta/OR (95% CI),** | **Beta/OR (95% CI),** |
| Systolic BP (mmHg) (Beta) | 2.19 (0.25 to 4.12) | 1.91 (-0.53, 4.34) |
| Diastolic BP (mmHg) (Beta) | 0.94 (-0.43 to 2.31) | 0.85 (-0.93, 2.62) |
| BMI (kg/m^2^) (Beta) | 1.14 (0.42 to 1.87) | 1.65 (0.73, 2.57) |
| WHR (Beta) | 0.01 (-0.00 to 0.02) | 0.01 (-0.00, 0.03) |
| Hypertension (OR) | 1.38 (1.00 to 1.89) | 1.31 (0.87, 1.97) |
| Overweight/obese vs. normal/underweight (OR) | 1.37 (1.02 to 1.82) | 1.63 (1.13, 2.36) |
| Moderate/high abdominal obesity vs normal (OR) | 1.29 (0.94 to 1.79) | 1.47 (0.97, 2.21) |
| ^a^ Adjusted for age, sex, center, education level, civil status, physical activity, smoking status, alcohol consumption, country of origin, and season.  ^c^ Never worked night shifts is the reference (n=175).  CI – confidence interval. | | |
