# Supplementary material for: Metabolic biomarkers and cardiometabolic risk among night shift workers: evidence from night shift workers in Europe
Source: Eur J Public Health. 2026 Jul 9;36(4):ckag101. doi: 10.1093/eurpub/ckag101 (PMC13348705; doi:10.1093/eurpub/ckag101)
Supplement: ckag101_Supplementary_Data [file ckag101_supplementary_data.zip › ejph-2026-01-om-0036-File008.docx]

Supplemental Table 7: Associations between plasma metabolites and cardiometabolic risk factors stratified by night versus day shift ^a^

|  | | | | | | | |
| --- | --- | --- | --- | --- | --- | --- | --- |
|  | **BMI** | **WHR** | **Overweight** | **Abdominal**  **Obesity** | **Systolic**  **BP** | **Diastolic**  **BP** | **Hypertension** |
|  | Beta (95% CI),  *p-value*^a^ | Beta (95% CI),  *p-value*^a^ | OR (95% CI),  *p-value*^a^ | OR (95% CI),  *p-value*^a^ | Beta (95% CI),  *p-value*^a^ | Beta (95% CI),  *p-value*^a^ | OR (95% CI),  *p-value*^a^ |
| Docosahexaenoic acid |  |  |  |  |  |  |  |
| Day shift | -14.57 (-22.50, -6.63),  <0.001 | -0.12 (-0.23, -0.01),  0.04 | 0.02 (0.001, 0.70), 0.03 | 0.01 (0.001, 0.84), 0.04 | 1.19 (-20.60, 22.98),  0.91 | -4.70 (-21.11, 11.72),  0.57 | 0.06 (0.001, 7.35),  0.25 |
| Night shift | -19.73 (-28.13, -11.33),  <0.001 | -0.14 (-0.27, -0.01),  0.03 | 0.001 (0.001, 0.05),  <0.001 | 0.15 (0.003, 8.47),  0.35 | -19.48 (-40.82, 1.86), 0.07 | -17.76 (-33.39, -2.12),  0.03 | 2.86 (0.01, 733.14),  0.71 |
| Omega-3 fatty acids to total fatty acids |  |  |  |  |  |  |  |
| Day shift | -0.52 (-1.02, -0.01),  0.05 | -0.004 (-0.01, 0.003),  0.24 | 0.83 (0.66, 1.04),  0.10 | 0.80 (0.61, 1.04),  0.10 | 0.33 (-1.05, 1.70),  0.64 | 0.07 (-0.97, 1.11),  0.90 | 1.11 (0.78, 1.58),  0.32 |
| Night shift | -0.82 (-1.33, -0.31),  <0.001 | -0.005 (-0.01, 0.003),  0.20 | 0.76 (0.61, 0.93),  0.01 | 0.95 (0.75, 1.20),  0.65 | -1.11 (-2.39, 0.16),  0.09 | -1.41 (-2.34, -0.47),  <0.01 | 0.92 (0.67, 1.28),  0.63 |
| Omega-6 fatty acids to total fatty acids |  |  |  |  |  |  |  |
| Day shift | -0.47 (-0.65, -0.28),  <0.001 | -0.003 (-0.006, -0.001),  0.01 | 0.84 (0.76 o 0.92),  <0.001 | 0.94 (0.85, 1.05),  0.27 | -0.47 (-0.99, 0.05),  0.07 | -0.44 (-0.83, -0.05),  0.03 | 0.92 (0.80, 1.04),  0.18 |
| Night shift | -0.55 (-0.72, -0.38),  <0.001 | -0.003 (-0.01, -0.001),  0.02 | 0.84 (0.77, 0.91),  <0.001 | 0.88 (0.80, 0.96),  <0.01 | -0.59 (-1.03, -0.15),  0.01 | -0.60 (-0.92, -0.28),  <0.001 | 0.88 (0.79, 0.98),  0.02 |
| Polyunsaturated fatty acids to total fatty acids |  |  |  |  |  |  |  |
| Day shift | -0.54 (-0.72, -0.35),  <0.001 | -0.004 (-0.007, -0.001).  <0.01 | 0.81 (0.73, 0.90),  <0.001 | 0.91 (0.81, 1.01),  0.08 | -0.42 (-0.94, 0.10),  0.11 | -0.42 (-0.81, -0.04),  0.03 | 0.93 (0.82, 1.06),  0.26 |
| Night shift | -0.64 (-0.80, -0.48),  <0.001 | -0.004 (-0.006, -0.001),  <0.01 | 0.80 (0.74, 0.87),  <0.001 | 0.87 (0.80, 0.95),  <0.01 | -0.71 (-1.15, -0.28),  <0.01 | -0.76 (-1.07, -0.44),  <0.001 | 0.87 (0.79, 0.97),  0.01 |
| Monounsaturated fatty acids to total fatty acids |  |  |  |  |  |  |  |
| Day shift | 0.76 (0.56, 0.97),  <0.001 | 0.01 (0.003, 0.009),  <0.001 | 1.38 (1.23, 1.56),  <0.001 | 1.16 (1.03, 1.31),  0.02 | 0.63 (0.04, 1.22),  0.04 | 0.57 (0.13, 1.01),  0.01 | 1.04 (0.89, 1.22),  0.63 |
| Night shift | 0.91 (0.71, 1.10),  <0.001 | 0.01 (0.003, 0.01),  <0.001 | 1.42 (1.27, 1.58),  <0.001 | 1.34 (1.19, 1.51),  <0.001 | 0.80 (0.27, 1.33),  <0.01 | 0.97 (0.59, 1.35),  <0.001 | 1.17 (1.02, 1.34),  0.02 |
| Saturated fatty acids to total fatty acids |  |  |  |  |  |  |  |
| Day shift | -0.13 (-0.46, 0.19),  0.43 | -0.001 (-0.01, 0.003),  0.71 | 0.93 (0.80, 1.07), 0.31 | 0.94 (0.79, 1.12),  0.51 | -0.18 (-1.05, 0.70),  0.69 | -0.03 (-0.69, 0.63),  0.93 | 0.92 (0.77, 1.10),  0.37 |
| Night shift | 0.12 (-0.21, 0.44),  0.48 | -0.002 (-0.01, 0.003), 0.38 | 0.97 (0.86, 1.10), 0.65 | 0.86 (0.74, 1.01),  0.06 | 0.61 (-0.19, 1.41),  0.14 | 0.36 (-0.23, 0.95),  0.23 | 1.16 (0.94, 1.43),  0.17 |
| Docosahexaenoic acid to total fatty acids |  |  |  |  |  |  |  |
| Day shift | -3.31 (-4.35, -2.26),  <0.001 | -0.03 (-0.05, -0.02), <0.001 | 0.29 (0.17, 0.50), <0.001 | 0.31 (0.16, 0.59), <0.001 | -23.96 (-5.93, 0.001), 0.05 | -2.87 (-5.09, -0.64),  0.01 | 0.49 (0.25, 0.94),  0.03 |
| Night shift | -4.52 (-5.57, -3.47),  <0.001 | -0.03 (-0.05, -0.02),  <0.001 | 0.16 (0.09, 0.27), <0.001 | 0.30 (0.16, 0.54),  <0.001 | -4.80 (-7.59, -2.00), <0.001 | -5.67 (-7.68, -3.67),  <0.001 | 0.64 (0.31, 1.32),  0.23 |
| Polyunsaturated fatty acids to monounsaturated fatty acids |  |  |  |  |  |  |  |
| Day shift | -5.58 (-7.19, -3.97),  <0.001 | -0.04 (-0.06, -0.01),  <0.01 | 0.10 (0.04, 0.24),  <0.001 | 0.40 (0.16, 0.98),  0.04 | -4.75 (-9.37, -0.12),  0.04 | -4.22 (-7.69, -0.75).  0.02 | 0.59 (0.16, 2.24),  0.44 |
| Night shift | -7.11 (-8.72, -5.51),  <0.001 | -0.05 (-0.07, -0.02),  <0.001 | 0.07 (0.03, 0.16),  <0.001 | 0.13 (0.05, 0.31),  <0.001 | -6.92 (-11.23, -2.61),  <0.01 | -8.26 (-11.36, -5.16),  <0.001 | 0.23 (0.07, 0.75),  0.02 |
| Linoleic acid to total fatty acids |  |  |  |  |  |  |  |
| Day shift | -0.41 (-0.61, -0.21),  <0.001 | -0.003 (-0.01, -0.001),  0.02 | 0.89 (0.81, 0.98), 0.02 | 0.96 (0.86, 1.07),  0.48 | -0.12 (-0.67, 0.43), 0.67 | -0.26 (-0.67, 0.15),  0.23 | 1.00 (0.89, 1.12),  0.96 |
| Night shift | -0.55 (-0.73, -0.36),  <0.001 | -0.002 (-0.01, 0.001),  0.11 | 0.87 (0.81, 0.95),  <0.001 | 0.94 (0.85, 1.03),  0.19 | -0.54 (-1.02, -0.06),  0.03 | -0.38 (-0.74, -0.03),  0.03 | 0.91 (0.81, 1.03),  0.12 |
| Alanine |  |  |  |  |  |  |  |
| Day shift | -0.09 (-6.14, 5.95),  0.98 | 0.02 (-0.07, 0.11),  0.65 | 0.54 (0.04, 7.80),  0.65 | 5.12 (0.19, 139.43),  0.33 | 7.85 (-8.39, 24.09),  0.34 | 5.90 (-6.32, 18.13), 0.34 | 2.04 (0.02, 169.97),  0.75 |
| Night shift | 5.21 (-1.37, 11.78),  0.12 | 0.06 (-0.04, 0.16),  0.23 | 16.70 (1.16, 240.49),  0.04 | 37.43 (1.30, 999.32),  0.03 | -1.72 (-18.15, 14.71),  0.84 | 5.34 (-6.69, 17.38),  0.38 | 0.06 (0.001, 5.40),  0.22 |
| Glycine |  |  |  |  |  |  |  |
| Day shift | -12.63 (-19.61, -5.65),  <0.001 | -0.13 (-0.23, -0.02),  0.01 | 0.01 (0.001, 0.24),  <0.01 | 0.06 (0.002, 2.05),  0.12 | -23.99 (-42.97, -5.01),  0.01 | -17.96 (-32.25, -3.67),  0.01 | 0.004 (0.001, 1.95),  0.08 |
| Night shift | -6.07 (-14.83, 2.69),  0.17 | -0.18 (-0.31, -0.05),  0.01 | 0.12 (0.004, 3.62),  0.22 | 0.02 (0.001, 1.23),  0.06 | -21.39 (-43.17, 0.39),  0.05 | -11.74 (-27.74, 4.27),  0.15 | 0.02 (0.001, 6.11),  0.17 |
| Total branched-chain amino acids^a^ |  |  |  |  |  |  |  |
| Day shift | 5.08 (0.53, 9.63),  0.03 | 0.05 (-0.01, 0.12),  0.10 | 15.82 (1.82, 137.76),  0.01 | 9.62 (0.65, 143.29),  0.10 | 6.43 (-5.88, 18.74),  0.31 | 2.29 (-6.99, 11.57),  0.63 | 0.92 (0.04, 19.75),  0.95 |
| Night shift | 4.35 (0.75, 7.95),  0.02 | 0.07 (0.02, 0.13),  0.01 | 13.28 (2.69, 65.69),  <0.01 | 55.47 (5.76, 534.03),  <0.001 | 3.45 (-5.58, 12.48),  0.45 | 1.71 (-4.91, 8.34),  0.61 | 0.87 (0.07, 10.59),  0.91 |
| Isoleucine |  |  |  |  |  |  |  |
| Day shift | 24.1 (1.67, 46.59), 0.04 | 0.32 (-0.01, 0.64),  0.05 | 999.0 (2.04, 999.0), 0.04 | 999.0 (0.11, 999.0), 0.10 | 28.56 (-32.22, 89.34),  0.36 | 11.42 (-34.39, 57.23),  0.62 | 0.02 (0.001, 999.0),  0.57 |
| Night shift | 22.26 (4.23, 40.29),  0.02 | 0.37 (0.10, 0.64),  0.01 | 999.0 (26.36, 999.0),  0.01 | 999.0 (999.0, 999.0),  <0.001 | 15.50 (-29.74, 60.74), 0.50 | 3.39 (-29.79, 36.57),  0.84 | 0.18 (0.001, 999.0),  0.07 |
| Valine |  |  |  |  |  |  |  |
| Day shift | 11.43 (2.45, 20.42),  0.01 | 0.12 (-0.01, 0.25),  0.07 | 447.2 (6.34, 999.0), 0.01 | 91.45 (0.51, 999.0),  0.09 | 18.47 (-5.86, 42.79),  0.14 | 9.20 (-9.15, 27.55),  0.32 | 8.64 (0.04, 999.0),  0.44 |
| Night shift | 9.39 (2.12, 16.66),  0.01 | 0.15 (0.04, 0.26),  0.01 | 274.08 (10.89, 999.0),  <0.001 | 999.0 (47.25, 999.0),  <0.001 | 8.18 (-10.06, 26.43), 0.38 | 5.98 (-7.40, 19.36),  0.38 | 1.87 (0.01, 264.21),  0.80 |
| Phenylalanine |  |  |  |  |  |  |  |
| Day shift | 39.56 (-4.70, 83.81),  0.08 | 0.52 (-0.11, 1.15),  0.11 | 20.7 (0.05, 982.0),  0.05 | 999.0 (999.0, 999.0),  0.01 | -27.70 (-147.34, 91.94),  0.65 | -23.08 (-113.16, 67.00),  0.61 | - 1. (0.001, 999.0),   2. 0.24 |
| Night shift | 51.97 (7.68, 96.27), 0.02 | 0.61 (-0.06, 1.29),  0.07 | 999.0 (55.26, 999.0),  0.02 | 999.0 (0.001, 999.0),  0.23 | 23.72 (-87.39, 134.82),  0.68 | 4.10 (-77.37, 85.59),  0.92 | 0.001 (0.001, 999.0), 0.53 |
| ^a^ Adjusted for age, sex, center, education level, civil status, physical activity, smoking status, alcohol consumption, country of origin, and season. | | | | | | | |
