# Supplementary figures and images for: Metabolic biomarkers and cardiometabolic risk among night shift workers: evidence from night shift workers in Europe
Source: Eur J Public Health. 2026 Jul 9;36(4):ckag101. doi: 10.1093/eurpub/ckag101 (PMC13348705; doi:10.1093/eurpub/ckag101)

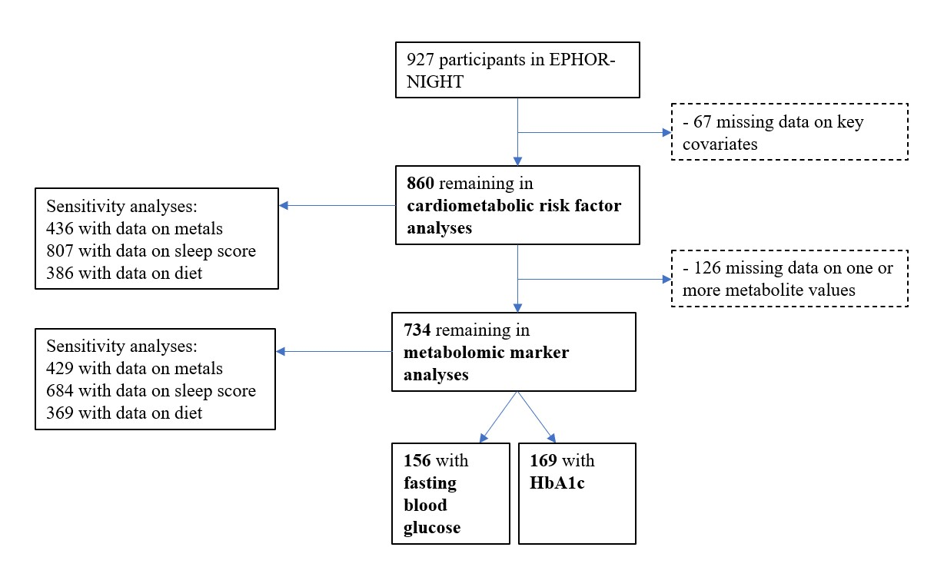

Supplement: ckag101_Supplementary_Data [file ckag101_supplementary_data.zip › ejph-2026-01-om-0036-File009.tif]

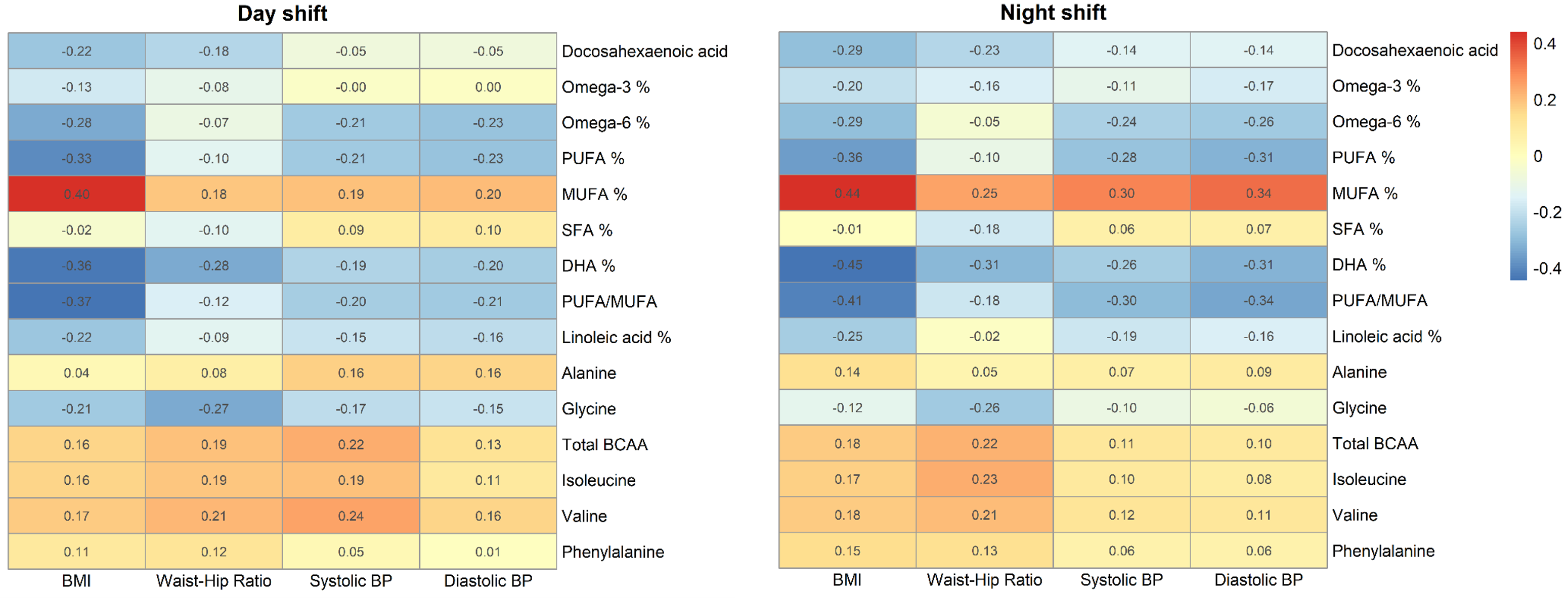

Supplement: ckag101_Supplementary_Data [file ckag101_supplementary_data.zip › ejph-2026-01-om-0036-File010.tif]
